# Genotype-Specific Modulatory Effects of Select Spectral Bandwidths on the Nutritive and Phytochemical Composition of Microgreens

**DOI:** 10.3389/fpls.2019.01501

**Published:** 2019-11-19

**Authors:** Marios C. Kyriacou, Christophe El-Nakhel, Antonio Pannico, Giulia Graziani, Georgios A. Soteriou, Maria Giordano, Armando Zarrelli, Alberto Ritieni, Stefania De Pascale, Youssef Rouphael

**Affiliations:** ^1^Department of Vegetable Crops, Agricultural Research Institute, Nicosia, Cyprus; ^2^Department of Agricultural Sciences, University of Naples Federico II, Portici, Italy; ^3^Department of Pharmacy, University of Naples Federico II, Naples, Italy; ^4^Department of Chemical Sciences, University of Naples Federico II, Naples, Italy

**Keywords:** amaranth, blue-red light, carotenoids, cress, minerals, mizuna, phenolic compounds, purslane

## Abstract

Advanced analytical data on microgreens' response to different light spectra constitutes a valuable resource for designing future crop-specific spectral management systems. The current study defined variation in productivity, nutritive and functional quality (mineral–carotenoid–polyphenolic profiles and antioxidant capacity) of novel microgreens (amaranth, cress, mizuna, purslane) in response to select spectral bandwidths (red, blue, blue-red), and appraised clustering patterns configured by the genotype-light-spectrum nexus. Growth parameters dependent on primary metabolism were most favored by blue-red light's efficiency in activating the photosynthetic apparatus. Nitrate accumulation was higher under monochromatic light owing to the dependency of nitrite reductase on the light-driven activity of PSI, most efficiently promoted by blue-red light. Although mineral composition was mostly genotype-dependent, monochromatic red and blue lights tended to increase K and Na and decrease Ca and Mg concentrations. Lutein, β-carotene, and lipophilic antioxidant capacity were generally increased by blue-red light putatively due to the coupling of heightened photosynthetic activity to increased demand for protection against oxidative stress; the disparate response however of purslane highlights the importance of genotype specificity in these responses and calls for additional investigation. Analysis of polyphenols by Orbitrap LC-MS/MS revealed substantial genotypic differences. Most abundant phenolics were chlorogenic acid ($\bar{x}$ = 5503 µg g^−1^ dw), feruloylquinic acid ($\bar{x}$ = 974.1 µg g^−1^ dw), and caffeoyl feruloyl tartaric acid ($\bar{x}$ = 993 µg g^−1^ dw). Hydroxycinnamic acids accounted for 79.0% of the mean total phenolic content across species, flavonol glycosides for 20.7%, and flavone glycosides for 0.3%. The general response across species was a decrease in individual polyphenolic constituents, particularly flavonol glycosides, and total polyphenols under blue-red light. The pronounced effectiveness of monochromatic blue light in eliciting synthesis of flavonoids could be linked to their capacity for absorbing shorter wavelengths thereby quenching generated photo-oxidation potential. The light-induced stimulation of the phenylpropanoid pathway by monochromatic blue light through epigenetic mechanisms or redox signaling in the photosynthetic apparatus warrants further investigation. The current work highlights how optimized genetic background combined with effective light management might facilitate the production of superior functional quality microgreens.

## Introduction

The promotion of healthy eating remains a topic of prime interest in modern societies (Kyriacou and Rouphael, 2018). As such, consumers are searching for potential nutrient-dense foods that may assist health and longevity (Kyriacou et al., 2016). Microgreens defined as tender immature greens are a specialty crop gaining popularity due to their fortified plant secondary metabolites (PSM) content, accumulated in their pair of first true leaves, compared to their mature-leaf counterparts (Xiao et al., 2012; Pinto et al., 2015; Xiao et al., 2015; Craver et al., 2017). In plant-rich diets, the PSM content imparts beneficial effects to human health, as PSM are known to play a primary role in delaying and/or inhibiting oxidative damage, thus preventing a range of common diseases like macular degeneration, cardiovascular diseases and cancer (Kennedy and Wightman, 2011; Khanam et al., 2012; Alrifai et al., 2019).

Although the content and composition of PSM in microgreens vary based on the genetic material (i.e., species), lots of factors are also implicated in modulating PSM, including cultural practices, conditions of cultivation, and environmental factors (Kyriacou et al., 2016; Kyriacou et al., 2017; Kyriacou et al., 2019a). As primary source of energy, light is one of the most important environmental factors along with air temperature for plant growth, development, and nutritional quality (Samuolienė et al., 2012; Wang et al., 2016). The appropriate lighting parameters (i.e., intensity, photoperiod, and spectral quality) can be optimized and modulated in high-tech greenhouses, plant factories, and controlled environment growth chambers using artificial lighting (Vaštakaitė et al., 2017; Rouphael et al., 2018). Compared with light intensity and duration, spectral quality shows much more complex responses in terms of crop productivity and functional quality, with mixed findings reported on microgreens (Alrifai et al., 2019).

Considering the importance of red and blue parts of the light spectra in several metabolic pathways and biological processes affecting the metabolism of bioactive compounds (phenolics, carotenoids, ascorbic acids, and tocopherols), the light-emitting diodes (LEDs) were introduced in plant cultivation at the beginning of this century as a more efficient light source compared to the most common high-pressure sodium lamps characterized by a high amount of orange-yellow light, with some red and low amount of blue or green spectral components (Son and Oh, 2015; Samuolienė et al., 2016; Samuolienė et al., 2017; Alrifai et al., 2019).

Plants have specialized receptors such as photosynthetic (carotenoid and chlorophyll) and photomorphogenetic (phytochromes and cryptochromes) light receptors, which are responsible for the photophysiological responses induced by light intensity and spectral quality changes, and also modulate several light-sensitive metabolomic/molecular pathways (Samuolienė et al., 2012; Samuolienė et al., 2013; Alrifai et al., 2019). Previous researchers have demonstrated that red light is sensed by phytochromes (PhyA to PhyE) and is responsible for the synthesis of phenolics and antioxidant activity. On the other hand, blue light is sensed by cryptochromes (CRY1 to CRY3) and is implicated in the biosynthesis of anthocyanins, ascorbic acid, chrlorophyll, and carotenoids (Li and Kubota, 2009; Olle and Viršilė, 2013; Ntagkas et al., 2018).

Over the past few years, spectral effects of red/blue/red-blue bandwidths on PSM have been investigated in microgreens species belonging to the families of *Brassicacceae* (mustard, kale, red pack choi, tatsoi, Kohlrabi, mizuna), *Lamiaceae* (basil and perilla), *Apiaceae* (parsley), *Boraginaceae* (borage), and *Chenopodiaceae* (beet, spinach) (Brazaitytė et al., 2015; Samuolienė et al., 2012; Samuolienė et al., 2016; Craver et al., 2017; Lobiuc et al., 2017). However, information on PSM profiles and how these bioactive compounds respond to LED spectral quality in new and emerging microgreens, like amaranth, cress, mizuna and purslane is missing. Since, there is ample evidence of species-specific response to the red/blue/red-blue spectral composition, there is an urgent need among scientists to understand the modulatory mechanism of red and blue light sensing on phytochemical profiles of emerging microgreens, that will definitely lead to the development of species-specific LEDs systems to boost yield and to improve important lipophilic and hydrophilic antioxidant compounds that could be beneficial to the human diet.

In perspective of the above considerations, the objectives of the current study were: i) to evaluate the nutritional and functional composition of new and emerging microgreens (amaranth, cress, mizuna, and purslane); ii) to understand the variation in productivity, mineral composition, antioxidant activity, target carotenoids, as well as qualitative/quantitative profiles of polyphenols in relation to the light spectra (red and blue LED light percentage); and iii) to appraise possible clustering patterns underlined by species and light quality interaction.

The results displayed in this paper will contribute to the understanding of spectral modulatory effects behind the variation in nutritional and functional quality of select microgreens in demand, and will assist the microgreens industry in identifying the optimum species-specific spectral quality systems for achieving fortified PSM content in select microgreens (Ntagkas et al., 2018). The current work may also contribute significantly to the pool of systematic information required to understand how the microgreens chemosphere is configured by the genotype-light-spectrum nexus and consequently how particular species may be streamlined for production under targeted optimal conditions.

## Materials and Methods

### Chemicals and Standards

Chicoric acid, chlorogenic acid, caffeic acid, catechin, epicatechin, rosmarinic acid, ferulic acid, rutin, vitexin, quercetin-3-*O*-glucoside, lutein, and β-carotene were obtained from Sigma (St. Louis, MO, USA). Quercetin-3-*O*-galactoside, kaempferol-7-*O*-glucoside, kaempferol-3-*O*-rutinoside, and 3,5-di-*O*-caffeoyl quinic acid were obtained from Extrasynthese (Genay, France). Methanol and formic acid (LC–MS grade) were obtained from Merck KGaA (Darmstadt, Germany). Ultrapure water was produced by a Milli-Q Gradient A10 water purification system. The purity of the standards was 98%, and all were prepared as initial stock solutions of 1 mg ml^−1^ in methanol. Lutein and β-carotene stocks of 1 mg ml^−1^ were prepared in chloroform. Multiple standards stock solutions were prepared as combinations of individual standard stock solutions with further dilutions made with methanol to obtain standard calibration curves in the range of 0.01–5.0 mg L^-1^.

### Plant Material and Growth-Chamber Conditions

Four microgreens species were assessed for their bioactive composition: mizuna (*Brassica rapa* var. *japonica* cv. Greens), amaranth (*Amaranthus tricolor* cv. Red garnet), cress (*Lepidium sativum* cv. Curled), and common purslane (*Portulaca oleracea* L.). Seeds of mizuna and amaranth were provided by Condor Seed Production (Yuma, Arizona, USA) while cress and purslane by Nehme Establishment for Trade & Agriculture (Batroun, Lebanon). The sowing density (i), 100-seed weight, (ii) and growth cycle duration, (iii) for mizuna, amaranth, cress, and purslane were respectively: (i) 7, 8, 6, and 8 seeds cm^−2^, (ii) 172.0, 75.0, 233.6, and 34.7 mg, and (iii) 16, 19, 20, and 21 days after sowing.

Experiments were conducted at the Department of Vegetable Crops of the Agricultural Research Institute, Nicosia, Cyprus, in controlled-environment Panasonic MIR-554 growth chambers (Panasonic, Gunma, Japan). Seeds were germinated in darkness at 24°C and 100% relative humidity. During the growing cycle day/night temperatures of 22/18 ± 2°C and RH of 65%–75% were established. Peat moss was chosen as growth substrate (pH: 6.3, EC: 0.2 dS m^−1^, porosity: 92% v/v and cation exchange capacity: 98 meq 100 g^−1^) to fill the plastic trays (14 cm × 19 cm × 6 cm: W × L × D). One growth chamber with an appropriately tuned LED panel was used to deliver each spectral treatment (red, blue or red-blue). In each chamber, three replicate seeding trays were used for each microgreens species.

For fertigation, a quarter-strength modified Hoagland formulation (2.0 mM NO_3_-N, 0.25 mM S, 0.20 mM P, 0.62 mM K, 0.75 mM Ca, 0.17 mM Mg, 0.25 mM NH_4_-N, 20 µM Fe, 9 µM Mn, 0.3 µM Cu, 1.6 µM Zn, 20 µM B, and 0.3 µM Mo) was adopted, accounting for an EC 0.4 ± 0.1 dS m^−1^ and pH 6 ± 0.2. Fertigation was applied manually by means of a laboratory wash bottle instead of foliar spraying (or overhead fertigation) in order to avoid excessive humidity on microgreens stems and leaves. Irrigation volume ranged between 50 and 200 ml/tray. The exact volume depended on the species, the growth stage, and the daily evapotranspiration of each tray monitored in terms of the weight loss of each tray between irrigation cycles.

**Figure 1:**
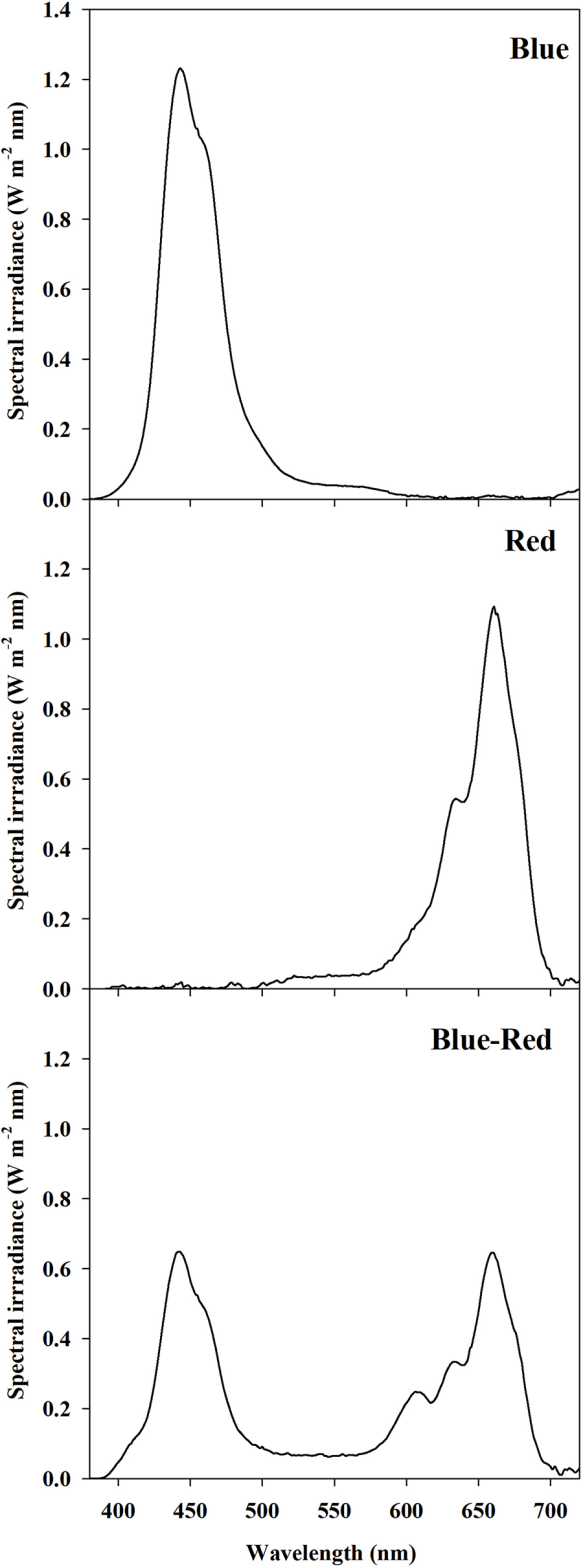
Spectral distribution of light treatments: red (90% R, 10% G, 0% B), blue (0% R, 10% G, 90% B), and red-blue (45% R, 10% G, 45% B). Spectra were recorded and averaged at twelve locations at the substrate level with a spectral radiometer. Photosynthetic photon flux density for each light treatment was equal to 300 ± 10 μmol m^−2^ s^−1^.

A 12 h photoperiod was provided by LED panel units (K5 Series XL750, Kind LED, Santa Rosa, CA, USA) with an emission wavelength range 400–700 nm divided into three customizable channels: red (R) (600–700 nm), blue (B) (400–500 nm), and green-yellow (G) (500–600 nm). The LED panel arrangement inside the growth chamber ensured full coverage of the entire surface of the canopy, providing a homogeneous photosynthetic photon flux density (PPFD) at the canopy level of 300 ± 10 µmol m^−2^ s^−1^. Moreover, the trays were arranged randomly and systematically rotated every 24 h to enhance the uniformity of the light environment. The PPFD and spectral composition were regulated at the beginning and confirmed at the end of each experimental replication run by twelve individual spectral scans per treatment using a spectral radiometer (MSC15, Gigahertz-Optik, Türkenfeld, Germany). Light treatments examined in the present experiment were: red (90% R, 10% G, 0% B), blue (0% R, 10% G, 90% B), and red-blue (45% R, 10% G, 45% B). The PPFD percentage contributions of R, G, and B were determined from bandwidth integration; the light spectrum of each treatment is reported in **Figure 1**.

### Sampling and Fresh Yield Assessment

Just after the appearance of the second true leaf, microgreens were clipped using sterilized scissors just above the substrate level. Fresh yield was determined for each tray and expressed in kg fw m^−2^. Determination of nitrate content, mineral composition, lipophilic antioxidant activity, and phenolic and carotenoid profiles was performed on fresh samples instantly frozen in liquid nitrogen, stored at −80°C before lyophilized in a Christ, Alpha 1-4 (Osterode, Germany) lyophilizer.

### Dry Matter, Nitrate, and Mineral Content Analysis

Dry matter content was determined on triplicate fresh samples following lyophilization to constant weight (≈48 h) measured on a Precisa XT120A analytical balance (Precisa Gravimetrics, Dietikon, Switzerland) and expressed as percentage of microgreens fresh mass. Dry matter samples used for chemical analyses were ground in a Wiley Mill to pass through an 841-microns screen. The concentrations of nitrate, P, K, S, Ca, Mg, and Na were determined following sample preparation and ion chromatography as previously described (Kyriacou et al., 2019a). Potassium, Ca, Mg, and Na were separated and quantified by ion chromatography (ICS-3000, Dionex, Sunnyvale, CA, USA) and electrical conductivity detection. Separation was achieved in isocratic mode on an IonPac CS12A analytical column (4 mm × 250 mm, Dionex, Corporation) equipped with an IonPac CG12A precolumn (4 mm × 250 mm, Dionex, Corporation) and a self-regenerating suppressor CERS500 (4 mm, Dionex, Corporation). The nitrate, P, and S anions were separated using an IonPac ATC-HC anion trap (9 mm × 75 mm) and an AS11-HC analytical column (4 mm × 250 mm) equipped with an AG11-HC precolumn (4 mm × 50 mm) and a self-regenerating suppressor AERS500 (4 mm). Nitrate concentration was converted to mg kg^−1^ fresh weight (fw) based on each sample's original content (dw), while P, K, S, Ca, Mg, and Na were expressed as g kg^−1^ dw.

### Assay of Lipophilic Antioxidant Activity

The lipophilic antioxidant activity of microgreens was assessed following extraction procedures previously described (Kyriacou et al., 2019a). Determination of the antioxidant activity in lipophilic extracts employed the 2,20-azinobis 3-ethylbenzothiazoline-6-sulfonic acid ABTS method (Pellegrini et al., 1999). Quantification relied on UV–Vis spectrophotometry, with the absorbance of lipophilic extracts measured at 734 nm. Trolox was used as the external standard to construct a calibration curve based on six concentration levels (2–20 µmol ml^−1^) with regression coefficient R^2^ > 0.99. Lipophilic antioxidant activity was expressed in mmol Trolox equivalent (6-hydroxy-2,5,7,8-tetramethylchro man-2-carboxylic acid) per 100 g dw.

### Separation and Quantification of Carotenoids by HPLC-DAD

Carotenoids were extracted from lyophilized samples in ethanol containing 0.1% BHT using a modification of the method of Kim et al. (2008) detailed in Kyriacou et al. (2019a). Separation of carotenoid molecules was performed using a Shimadzu HPLC Model LC 10 (Shimadzu, Osaka, Japan) equipped with a reverse phase 250 mm × 4.6 mm, 5 µm Gemini C18 column (Phenomenex, Torrance, CA, USA). Injection volume per sample was 20 µl. Acetonitrile (mobile phase A) and ethanol:n-hexane:dichloromethane (1:1:1; mobile phase B) were used to build the following A:B gradient: 0–8 min (82:18); 8–12 min (76:24); 12–18 min (39:61); and 18–25 min a linear gradient to equilibration (82:18). Total runtime was 25 min. Absorbance was measured at 450 nm. Quantification was performed against linear calibration curves built with lutein and β-carotene external standards including at least six levels of concentration ranging 5 to 100 µg ml^−1^; both were expressed as mg kg^-1^ dw.

### Extraction and Analysis of Polyphenols by UHPLC-Q-Orbitrap HRMS

Polyphenols were extracted from lyophilized microgreens (100 mg) using 5 ml methanol/water (60:40, v/v) and sonication for 30 min at room temperature. Suspensions were centrifuged at room temperature for 15 min at 4000 rpm and then filtered through a 0.45 µm filter paper (Whatman International Ltd., Maidstone, U.K.). Ten microliters of the filtrate were used for mass spectrometry (HRMS-Orbitrap) analysis.

Separation and quantification of polyphenols was performed on an UHPLC system (UHPLC, Thermo Fisher Scientific, Waltham, MA, USA) equipped with a Dionex Ultimate 3000 Quaternary pump performing at 1250 bar and a thermostated (T = 25°C) Kinetex 1.7 µm biphenyl (100 mm × 2.1 mm) column (Phenomenex, Torrance, CA, USA). An injection volume of 2 µl was used and eluted at a flow rate of 0.2 ml min^−1^ using a gradient of (A) 0.1% formic acid in H_2_O, and (B) 0.1% formic acid in methanol as follows: 0 min—5% B, 1.3 min—30% B, 9.3 min—100% B, 11.3 min—100% B, 13.3 min—5% B, and 20 min—5% B.

Mass spectrometry analysis was facilitated by a Q Exactive Orbitrap LC-MS/MS (Thermo Fisher Scientific, Waltham, MA, USA). All compounds were analyzed using an ESI source (HESI II, Thermo Fischer Scientific, Waltham, MA, USA) in negative ion mode (ESI−) with −2.8 kV spray voltage, sheath gas (N_2_ > 95%) 45, auxiliary gas (N_2_ > 95%) 10, capillary temperature 275°C, S-lens RF level 50, and auxiliary gas heater temperature 305°C. Acquisition of polyphenolic compounds was carried out on parallel reaction monitoring mode, with settings as previously described (Kyriacou et al., 2019a). Input time frame for elution and collision energy were optimized for each polyphenolic compound. A Thermo Fisher Scientific reference standard mixture was used to monitor the accuracy and calibration of the Q Exactive Orbitrap LC-MS/MS. The mass tolerance window was set to 5 ppm for the two analysis modes. Six concentration levels were used to assess linearity at the low [limit of quantitation (LOQ)—5 mg kg^−1^] and six at the high (5–120 mg kg^−1^) concentration range. The limits of detection (LODs) and LOQs of the methods were determined. The LOD and LOQ values for the LC-MS/MS analysis of polyphenols were based on chlorogenic acid and rutin signal-to-noise levels. In the case of HPLC-DAD analysis for carotenoids, the LOD and LOQ values were determined for β-carotene. LOD and LOQ for each compound were obtained by serial dilutions of stock solution. Analysis and processing of data were performed using the Xcalibur software, v. 3.0.63 (Xcalibur, Thermo Fisher Scientific).

### Statistics

Two-way ANOVA was performed on the experimental data using the SPSS 20 software package. Treatment means were compared using Duncan's Multiple Range Test performed at P ≤0.05. In order to explore relationships among variables and treatments principal component analysis (PCA) was performed (A) on yield, mineral composition, carotenoid composition, and total phenolics, and (B) on 13 key phenolic compounds and on total phenolic content of the four microgreens species treated with three light conditions. PCA was performed using the SPSS 20 software package.

## Results and Discussion

### Fresh Biomass Yield and Dry Matter Content

The growth period from sowing to full harvest maturity, corresponding to the second true leaf stage, varied considerably for mizuna, amaranth, cress, and purslane microgreens, being respectively 16, 19, 20, and 21 days after sowing. Overall, the observed growth periods exceeded those reported for the same species in previous works where harvesting was performed at an earlier (cotyledonary or first true leaf) stage (Pinto et al., 2015; Xiao et al., 2016; Di Gioia et al., 2017).

**Figure 2:**
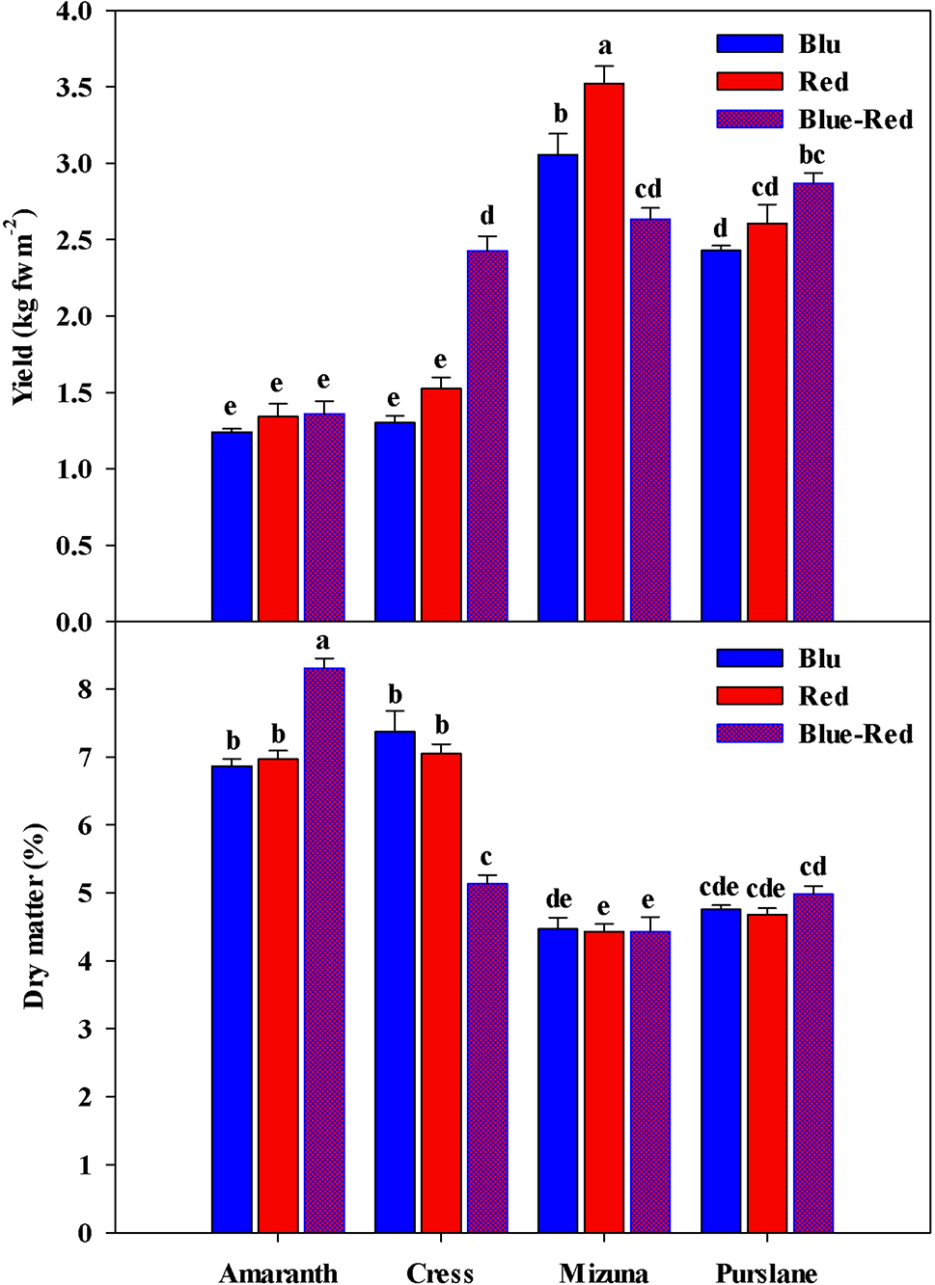
Fresh yield and dry matter of four microgreens genotypes as modulated by variable spectral bandwidths in a controlled growth environment. All data are expressed as mean ± standard error, n = 3.

Fresh biomass yield varied between species (**Figure 2**), with the highest fresh yield obtained from mizuna (2.63–3.52 kg m^−2^), followed by purslane (2.43–2.87 kg m^−2^), cress (1.30–2.43 kg m^−2^), and amaranth (1.24–1.36 kg m^−2^). In terms of dry mater content however (**Figure 2**), species ranking was inversed, as it was highest in amaranth (6.86%–8.31%), followed by cress (5.13%–7.37%), purslane (4.68%–4.98%), and finally mizuna (4.43%–4.47%). The yield obtained across species was overall higher than previously reported (Di Gioia et al., 2017), owing likely to the higher seed density applied in the current work as well as to the longer growth cycle and more advanced growth stage attained before harvest. Regardless of the above overall differences observed between microgreens species with regards to fresh biomass yield and dry matter content, the significant interaction observed between microgreens species (M) and light treatment (L) constitutes an important finding of the present study. This interaction stems from the differential response of the four species to the light conditions applied. In terms of fresh biomass, amaranth demonstrated no response to light treatments (**Figure 2**), as opposed to the rest three species. Both cress and purslane demonstrated the highest fresh yield when grown under blue-red than under red or blue light alone. This type of response was expected since combined blue-red LED bandwidths correspond to the absorption spectra of chlorophyll a and b and have been shown to promote plant growth (Amoozgar et al., 2017). Blue light perception and signaling mediated by cryptochrome photo-receptors is believed to reduce cell wall extensibility and repress cell elongation (Huché-Thélier et al., 2016). However, in the case of mizuna microgreens the highest fresh yield was attained under red light, followed by blue light, while yield was significantly compromised under the blue-red treatment. Ohashi-Kaneko et al. (2007) reported a 39% and 58% reduction in lettuce and spinach dry matter content, respectively, when grown under blue as opposed to white light. The ability of monochromatic blue and red light to increase biomass is exceptional but has been previously demonstrated on basil (Samuoliene et al., 2016; Alrifai et al., 2019). The underlying mechanism of this species-specific response remains unresolved but is thought to implicate monochromatic light's effect on stomatal conductance and thereby on photosynthetic activity (Huché-Thélier et al., 2016). Two of the species responsive to light treatment (mizuna and purslane) in terms of fresh biomass yield, showed no differentiation as regards their dry matter content. Cress microgreens demonstrated the inverse response to light treatments in terms of fresh yield (blue-red > red = blue) and dry matter content (blue = red > blue-red). Finally, amaranth demonstrated higher dry matter content under the blue-red treatment compared to blue and red treatments alone. The differential growth responses of the four microgreens species possibly reflect blue light's species-specific action on the CRY gene expression, as previously manifested for instance in the downregulation of PsCRY2b expression in *P. sativum* L. and the enhancement of CRY1 expression in *Brassica napus* (Huché-Thélier et al., 2016). Important conclusions derived from the above results are: (a) combined blue-red bandwidths are generally more effective at increasing microgreens fresh biomass production than blue and red bandwidths alone, however species response to variable bandwidths may vary; (b) species responses to bandwidth in terms of fresh biomass yield and dry matter content are generally opposite; for instance, mizuna and purslane seem to readily expend photosynthates in order to fuel rapid cell division taking place at the meristematic regions of their microgreen seedlings, they take up water more efficiently to drive cell expansion and, consequently, they tend to accumulate less dry matter. Differential bandwidth effects on fresh yield and dry matter may reflect different bandwidth efficiencies in activating the photosystems of microgreens (Amoozgar et al., 2017).

### Nitrate Concentration and Mineral Composition

Fresh vegetable consumption is the primary source of nitrate intake, high levels of which have been associated with increased probability for carcinogenic nitrosamine formation in the stomach (EFSA, 2008; Colla et al., 2018; Kyriacou et al., 2019b). As a protective measure, the European Commission (EC), 2006 established maximum nitrate concentrations for three common leafy vegetables: lettuce (3000–5000 mg kg^−1^ fw); spinach (3500 mg kg^−1^ fw); and rocket (6000–7000 mg kg^−1^ fw) (EC, 2006). Little information is so far available for the nitrate levels in microgreens, which constitute an emerging fresh salad component attracting growing culinary and nutraceutical interest. Nitrate concentration among the four microgreens species presently studied varied significantly (**Table 1**). Cress (5074 ± 259 mg kg^−1^ fw) was the highest nitrate accumulator, amaranth (2386 ± 139 mg kg^−1^ fw) accumulated low concentrations of nitrate, whereas mizuna (2856 ± 191 mg kg^−1^ fw) and purslane (3499 ± 238 mg kg^−1^ fw) were moderate accumulators. Nitrate accumulation capacity is a trait strongly associated with plant genetic background and characterizes both mature vegetables and their microgreen counterparts (Kyriacou et al., 2016). Genetic variation underlines differences in nitrate uptake efficiency, translocation capacity and vacuolar accumulation potential in the mesophyll cells (Blom-Zandstra, 1989). In this respect, the *Brassicaceae*, *Amaranthaceae*, *Lamiaceae*, and *Apiaceae* botanical families are the ones generally associated with high nitrate accumulation capacity (Colla et al., 2018; Kyriacou et al., 2019b). However, as indicated by the comparative nitrate levels presently found in cress and mizuna, both members of *Brasicaceae*, nitrate accumulation capacity in microgreens may be subject to considerable genetic variability even across genera of the same family. European Commission regulation (EU) No 1258/2011 defined the maximum allowable levels of nitrate in spinach at 3500 mg kg^−1^ fw, in summer/winter lettuce grown under cover at 5000/4000 mg kg^−1^ fw, and in summer/winter rucola (a known hyper-accumulator from the *Brasicaceae* family) at 7000/6000 mg kg^−1^ fw. Although the four microgreens species examined are not yet regulated with respect to nitrate concentration, moreover microgreens are generally consumed in lower amounts than their mature-leaf counterparts, it is apparent from the present results that the range of concentrations potentially encountered in microgreens can be high, as for instance in cress (4086–5562 mg kg^−1^ fw). Notwithstanding the genetic determinants of nitrate levels in microgreens, several approaches effective at reducing nitrate have been described in a previous works (Colla et al., 2018). These approaches include the reduction of nitrate concentration in the nutrient solution and switching to a nitrate-free solution in order to implement nitrate-starvation shortly before harvest.

**Table 1:** Nitrate, phosphorus (P), potassium (K), calcium (Ca), magnesium (Mg), sulfur (S), and sodium (Na) concentrations of four microgreens genotypes as modulated by variable spectral bandwidths in a controlled growth environment.

Source of variance	Nitrate (mg kg^−1^ fw)	P (g kg^−1^ dw)	K (g kg^−1^ dw)	Ca (g kg^−1^ dw)	Mg (g kg^−1^ dw)	S (g kg^−1^ dw)	Na (g kg^−1^ dw)
Microgreens species (M)							
Amaranth	2386 ± 139 d	6.48 ± 0.32 c	47.01 ± 1.72 c	2.49 ± 0.13 c	20.17 ± 0.94 a	4.22 ± 0.39 c	0.66 ± 0.08 c
Cress	5074 ± 259 a	8.27 ± 0.19 b	52.13 ± 0.89 b	16.10 ± 0.47 b	5.30 ± 0.26 d	11.99 ± 0.57 a	3.53 ± 0.28 b
Mizuna	2856 ± 191 c	6.43 ± 0.21 c	43.23 ± 1.02 d	25.56 ± 0.67 a	7.07 ± 0.16 c	9.83 ± 0.23 b	12.02 ± 1.26 a
Purslane	3499 ± 238 b	10.08 ± 0.55 a	62.99 ± 2.05 a	0.59 ± 0.04 d	15.28 ± 1.47 b	3.16 ± 0.18 d	3.15 ± 0.34 b
Light quality (L)							
Blue	3893 ± 347 a	8.33 ± 0.62 a	54.52 ± 2.39 a	11.23 ± 3.10 a	11.02 ± 1.72 b	7.47 ± 1.39 a	5.08 ± 1.61 a
Red	3436 ± 424 b	7.83 ± 0.61 a	52.38 ± 2.80 a	10.45 ± 2.84 b	10.40 ± 1.65 b	7.57 ± 1.20 a	5.29 ± 1.68 a
Blue-Red	3032 ± 212 c	7.29 ± 0.26 b	47.13 ± 1.95 b	11.88 ± 3.35 a	14.43 ± 2.28 a	6.87 ± 0.81 b	4.16 ± 0.73 b
M × L							
Amaranth × Blue	2647 ± 152 def	6.97 ± 0.27 def	50.50 ± 2.33	2.75 ± 0.09 e	19.13 ± 1.54 bc	2.92 ± 0.14 ef	0.81 ± 0.02 e
Amaranth × Red	2125 ± 248 f	6.16 ± 0.61 ef	47.28 ± 2.17	2.68 ± 0.15 e	18.68 ± 1.25 c	4.94 ± 0.22 d	0.75 ± 0.17 e
Amaranth × Blue-Red	2386 ± 281 ef	6.31 ± 0.75 ef	43.26 ± 3.50	2.05 ± 0.13 ef	22.69 ± 1.33 a	4.81 ± 0.72 d	0.43 ± 0.03 e
Cress × Blue	5562 ± 167 a	8.89 ± 0.20 b	53.63 ± 0.74	15.37 ± 0.43 d	4.80 ± 0.05 f	13.43 ± 0.49 a	2.75 ± 0.19 d
Cress × Red	5572 ± 21 a	8.26 ± 0.20 bc	53.66 ± 1.24	15.17 ± 0.20 d	4.76 ± 0.07 f	12.63 ± 0.46 a	3.27 ± 0.17 d
Cress × Blue-Red	4086 ± 214 b	7.67 ± 0.05 cd	49.09 ± 0.71	17.77 ± 0.56 c	6.34 ± 0.14 ef	9.92 ± 0.08 bc	4.57 ± 0.18 c
Mizuna × Blue	3215 ± 401 cd	6.05 ± 0.08 f	46.79 ± 0.72	26.09 ± 0.53 a	6.95 ± 0.03 ef	10.43 ± 0.21 b	14.19 ± 0.51 a
Mizuna × Red	2338 ± 252 ef	6.03 ± 0.21 f	42.50 ± 0.67	23.50 ± 0.82 b	6.62 ± 0.15 ef	10.05 ± 0.18 b	14.74 ± 0.81 a
Mizuna × Blue-Red	3015 ± 48 de	7.22 ± 0.13 cde	40.41 ± 0.96	27.10 ± 1.03 a	7.63 ± 0.15 e	9.01 ± 0.15 c	7.14 ± 0.29 b
Purslane × Blue	4149 ± 103 b	11.40 ± 0.17 a	67.14 ± 1.06	0.69 ± 0.06 fg	13.23 ± 0.30 d	3.08 ± 0.23 ef	2.55 ± 0.04 d
Purslane × Red	3708 ± 211 bc	10.86 ± 0.42 a	66.07 ± 3.01	0.46 ± 0.05 g	11.53 ± 0.09 d	2.67 ± 0.26 f	2.42 ± 0.16 d
Purslane × Blue-Red	2641 ± 153 def	7.98 ± 0.23 bcd	55.76 ± 0.94	0.61 ± 0.05 g	21.08 ± 0.06 ab	3.74 ± 0.05 e	4.48 ± 0.10 c
Significance							
Microgreens species (M)	***	***	***	***	***	***	***
Light quality (L)	***	**	***	***	***	*	***
M × L	***	***	ns	***	***	***	***

All data are expressed as mean ± standard error, n = 3.

ns, *, **, *** Nonsignificant or significant at P ≤ 0.05, 0.01, and 0.001, respectively. Different letters within each column indicate significant differences according to Duncan's multiple-range test (P = 0.05).

Aside from the effect of plant genotype, nitrate levels in microgreens were significantly affected by light bandwidth (**Table 1**). The highest nitrate accumulation (3893 ± 347 mg kg^−1^ fw) was observed under blue light, followed by red (3436 ± 424 mg kg^−1^ fw) and blue-red light (3032 ± 212 mg kg^−1^ fw). However, significant M × L interaction was observed as bandwidth effects were not uniform across species. The blue > red > blue-red effect on nitrate accumulation was more evident on the higher-accumulating species cress and purslane and less so on amaranth and mizuna. However, the general light spectral effect observed was that combined blue-red light was more effective at promoting nitrate assimilation, thus resulting in lower nitrate residual concentrations in microgreens. The capacity of combined blue-red bandwidth to reduce nitrate accumulation likely relates to the higher photosynthetic activity sustained under combined bandwidths that in turn furnishes the carbon and energy necessary for nitrogen assimilation (Champigny, 1995). Combined blue-red bandwidths may maximize the efficiency of Photosystem I that is necessary to drive nitrate reduction (Riens and Heldt, 1992; Ohashi-Kaneko et al., 2007; Qi et al., 2007; Kyriacou et al., 2016; Colla et al., 2018). Nitrate assimilation in photosynthetic cells is dependent on the tandem reduction of nitrate and nitrite ions. Nitrate reduction takes place in the cytosol through the NADH-specific or NAD(P)H-bispecific action of nitrate reductase and nitrite is subsequently transported to the chloroplast stroma wherein it is further reduced to ammonium by the activity of nitrite reductase (Riens and Heldt, 1992). Nitrite reductase activity is largely light-dependent, driven by the function of PS I, however limited nitrite reduction may rely on reducing agents supplied by catabolic pathways (Colla et al., 2018). Under dark conditions nitrite reductase activity is halted and nitrate reductase activity is also arrested, possibly through feedback inhibition so as to avoid cytotoxic nitrite accumulation (Riens and Heldt, 1992). However, the exact mechanism coordinating the activity of nitrate reductase in the cytosol with photosynthetic activity in the chloroplasts remains largely unresolved (Colla et al., 2018).

The mineral profile of microgreens species has yet received limited attention and existing information from previous studies is unreconciled due to differences in cultivation parameters including growth substrate, nutrient supply and developmental stage at harvest (Xiao et al., 2016). Harvesting prior to the developmental stage defining microgreens, i.e. the second true leaf stage (Kyriacou et al., 2016), is liable to introduce significant variation to the mineral composition profile of the products, as previously demonstrated during stages of seedlings ontogeny (Pinto et al., 2015). With respect to the concentrations of macronutrients P, K, Ca, Mg, S, and Na significant variation was observed among the four microgreens species examined (**Table 1**). Genotypic differences were most pronounced in terms of Ca (0.59–25.56 g kg^−1^ dw), Mg (5.30–20.17 g kg^−1^ dw), S (3.16–11.99 g kg^−1^ dw), and Na (0.66–12.02 g kg^−1^ dw). More limited genotypic differences were observed with respect to the concentrations of P (6.43–10.08 g kg^−1^ dw) and K (43.23–62.99 g kg^−1^ dw). Purslane exhibited the highest concentrations of P and K, mizuna of Ca and Na, and amaranth of Mg. Both cress and mizuna exhibited distinctly high concentrations of S, which is a signature trait of the glucosinolate-rich *Brassicaceae* microgreens (Neugart et al., 2018). Another important differentiation of genotypes was observed with respect to the Na/K ratio which was notably higher in mizuna (0.28) compared to the rest of the species examined (0.01–0.07). This is a trait of particular interest for human nutrition given the association of low Na/K foods with a lower incidence of high blood pressure and cardiac arrest (Choi et al., 2005).

The incidence of significant M × L interaction indicates differential response to light treatments of the four microgreens species examined with respect to mineral concentrations (**Table 1**). However, the relative contribution of the main effects and their interaction to the variance of mineral concentrations (variance breakdown not shown) indicates that variation is introduced principally by microgreens genotype (M) and much less so by light treatment (L) or the M × L interaction. The effect of light treatment was more pronounced and unambiguous on K and Na concentrations, both of which were significantly higher under the red and blue treatments than under the blue-red light treatment, whereas a generally opposite effect was observed on Ca and Mg concentrations.

### Antioxidant Activity and Carotenoid Content

The lipophilic fractions of the four microgreens species examined demonstrated widely variable antioxidant activities (**Table 2**). Antioxidant activity was highest in cress (92.6 ± 2.0 mmol Trolox eq. 100 g^–1^ dw) and lowest in amaranth (65.7 ± 4.1 mmol Trolox eq. 100 g^–1^ dw). However, purslane exhibited the highest concentrations of lutein (107.0 ± 7.2 mg kg^−1^ dw) and β-carotene (254.3 ± 16.1 mg kg^−1^ dw). Both of these hydrophobic carotenoid molecules have shown to possess lipophilic antioxidant capacity owing mainly to the conjugated double bonds of their long polyene chain responsible for their light-absorbing properties and the quenching of reactive oxygen species formed during photosynthesis (Young and Lowe, 2001). As a supplement to human nutrition, lutein improves eye protection against short wavelengths, light-induced oxidative damage, and macular degeneration (Kvansakul et al., 2006). Beta-carotene demonstrates biological activity as a precursor of vitamin A (Provitamin A) that is essential for growth, immune function, and vision.

**Table 2:** Lipophilic antioxidant activity (LAA), lutein, and β-carotene concentrations of four microgreens genotypes as modulated by variable spectral bandwidths in a controlled growth environment.

Source of variance	LAA (mmol Trolox eq. 100 g^–1^ dw)	Lutein (mg kg^−1^ dw)	β-carotene (mg kg^−1^ dw)
Microgreens species (M)			
Amaranth	65.67 ± 4.1 c	97.2 ± 9.8 b	214.2 ± 24.6 b
Cress	92.58 ± 2.0 a	95.2 ± 7.5 b	218.0 ± 22.3 b
Mizuna	85.91 ± 2.6 b	68.8 ± 5.0 c	164.6 ± 11.8 c
Purslane	84.23 ± 2.6 b	107.0 ± 7.2 a	254.3 ± 16.1 a
Light quality (L)			
Blue	79.36 ± 2.9 b	78.0 ± 3.4 c	198.3 ± 13.1 b
Red	78.92 ± 5.0 b	88.6 ± 9.0 b	186.4 ± 20.4 c
Blue-Red	88.02 ± 2.8 a	109.6 ± 6.4 a	253.6 ± 16.6 a
M × L			
Amaranth × Blue	63.79 ± 1.4 f	79.7 ± 0.5 d	186.4 ± 1.5 de
Amaranth × Red	52.63 ± 1.3 g	76.0 ± 1.4 d	146.7 ± 4.7 h
Amaranth × Blue-Red	80.60 ± 0.5 e	135.9 ± 5.0 a	309.5 ± 4.1 a
Cress × Blue	87.76 ± 1.5 cd	73.9 ± 1.9 d	170.7 ± 2.7 fg
Cress × Red	90.86 ± 2.6 bc	88.7 ± 2.1 c	176.5 ± 5.0 ef
Cress × Blue-Red	99.13 ± 2.2 a	123.1 ± 4.8 b	306.9 n± 2.6 a
Mizuna × Blue	85.42 ± 1.2 d	64.1 ± 0.7 e	164.4 ± 0.6 g
Mizuna × Red	77.69 ± 0.7 e	54.4 ± 1.7 f	123.9 ± 1.9 i
Mizuna × Blue-Red	94.62 ± 2.2 b	87.8 ± 2.3 c	205.4 ± 1.6 c
Purslane × Blue	80.47 ± 0.9 e	94.3 ± 3.0 c	271.7 ± 8.3 b
Purslane × Red	94.49 ± 0.6 b	135.2 ± 3.1 a	298.6 ± 1.9 a
Purslane × Blue-Red	77.72 ± 0.5 e	91.5 ± 1.2 c	192.6 ± 1.7 d
Significance			
Microgreens species (M)	***	***	***
Light quality (L)	***	***	***
M × L	***	***	***

All data are expressed as mean ± standard error, n = 3.

*** Significant at P ≤ 0.001, respectively. Different letters within each column indicate significant differences according to Duncan's multiple-range test (P = 0.05).

Aside from genotypic differences in microgreens carotenoids content and lipophilic antioxidant activity, a significant M × L interaction was observed with respect to the above variables. This interaction derived from the differential response of the four species to light treatments. Three of the species examined (amaranth, cress, mizuna) demonstrated significantly higher lutein, β-carotene, and lipophilic antioxidant activity when grown under blue-red light than under either bandwidth alone (**Table 2**). Purslane by contrast had the highest lipophilic antioxidant activity and carotenoid content under monochromatic red light than under either blue or combined blue-red light. Such increase in antioxidant capacity in response to monochromatic light has been previously demonstrated with lettuce receiving supplementary or increased blue LED light, but the underlying mechanism of this induction and its genotype-specificity remain poorly understood (Johkan et al., 2010). Notwithstanding the genotype–bandwidth interaction, a general conclusion that may be derived from the current work is that combined blue-red light is generally more effective than monochromatic blue or red light in driving an increase in the carotenoid content and the lipophilic antioxidant capacity of most microgreens species. The increase in lutein and β-carotene concentrations and the concomitant increase in antioxidant capacity is likely linked to enhanced photosynthetic activity promoted by the combined bandwidths. Heightened activity of photosystem I could trigger the biosynthesis of additional light-quenching carotenoid molecules to provide protection from oxidative stress (Saltveit, 2010), as carotenoids have been shown to function as scavengers of reactive oxygen species and as quenchers of singlet oxygen molecules, thus protecting photosynthetic apparatus and membrane lipids against oxidative damage (Jahns and Holzwarth, 2012). Previous work has demonstrated that light intensity can influence chloroplast redox status and accumulation of carotenoids, in particular lutein, β-carotene and violaxanthin + neoxanthin, as well as antioxidant enzymes activity (Jahns and Holzwarth, 2012). The sharp increase in both carotenoids and antioxidant capacity observed under blue-red light in three of the four species examined highlights the potential use of spectral management for the production of microgreens fortified with antioxidant phytochemicals and enhanced in functional quality (Ntagkas et al., 2018). Further research is nonetheless warranted to investigate the specificity of such preharvest applications, including genotype-dependent responses, that may constitute valuable tools toward enhancing the nutraceutical value of this emerging functional food (Kyriacou et al., 2019a).

### Phenolic Profiles

Chromatographic separation and quantitation by Q Exactive Orbitrap LC-MS/MS analysis of the polyphenols extracted from the four microgreens species treated with variable spectral bandwidths, revealed substantial differences of genotypic origin. In terms of total polyphenols, calculated as the sum of the individual polyphenols quantitated, purslane had the highest content (13,581 ± 182 µg g^−1^ dw) followed closely by amaranth (12,825 ± 330 µg g^−1^ dw), whereas mizuna (5122 ± 212 µg g^−1^ dw) and cress (4354 ± 156 µg g^−1^ dw) had notably lower polyphenolic contents. Similar levels of polyphenols in microgreens were previously reported by Xiao et al. (2015; 1500–7000 µg g^−1^ dw) and by Bulgari et al. (2017; 164–328 µg g^−1^ fw), determined however by spectrophotometry using gallic acid as the external standard. The predominance of chlorogenic acid, which accounts for most of the total phenolic content presently found in purslane and amaranth microgreens, has been previously found in several *B. oleracea* L. cultivars at the seedling stage (Vallejo et al., 2003) and has been described as one of the most efficient and abundant antioxidant products of the phenylpropanoid pathway in young plant tissues (Saltveit, 2010).

Twelve principal phenolic compounds extracted from the four microgreens species examined in the present study were identified and quantified (**Table 3**). In terms of relative content (percentage of total phenolic content), the most abundant phenolic compounds across species were chlorogenic acid (998–10,125 µg g^−1^ dw), feruloylquinic acid (971–983 µg g^−1^ dw), and caffeoyl feruloyl tartaric acid (970–1056 µg g^−1^ dw). Overall, of the 12 principle phenolic compounds quantified, hydroxycinnamic acids and their derivatives accounted for 79.0% of the mean total phenolic content across species, flavonol glycosides for 20.7% and flavone glycosides for 0.3%. However, significant qualitative differences were identified between species with respect to their phenolic profiles. Significantly higher concentrations of signature phenolics were identified in certain species: apigeninmalonyl glucoside, caffeic acid, p-coumaric acid, and kaempferol-3-*O*-synapoil-sophoroside-7-*O*-glucoside were highest in mizuna microgreens; feruloylglycoside was highest in cress; whereas chlorogenic acid was distinctively higher in purslane and amaranth compared to cress and mizuna. Previous studies have reported on the relative abundance of flavonols (e.g., kaempferol, quercetin, and isorhamnetin glycosides) in mature vegetables of cruciferous species (Cartea et al., 2011; Li et al., 2018). However, the microgreens of cruciferous species examined in the present study were less abundant in flavonol glycosides, accounting for only 26.8% and 33.1% of polyphenols in cress and mizuna, respectively (**Table 3**). In fact, the phenolic profiles of all four species examined were dominated by hydroxycinnamic acids and their derivatives. This difference might be attributed to the dramatic compositional changes taking place during the developmental stages of vegetable species from seed to mature vegetable. Previous work profiling the phenolic composition of broccoli (*B. oleracea* L.) along progressive developmental stages has demonstrated the relative abundance of chlorogenic acid during the young seedling stage and its progressive decrease relative to the total flavonoids content, despite the rise in absolute concentration (Vallejo et al., 2003). Similarly, the phenolic profile of cress sprouts abounded in hydroxycinnamic acids and their derivatives, as compared to flavonoids (Oszmiański et al., 2013). Developmental transformation of phenolic compositional profile is apparently more pronounced for certain species than others; for instance, coriander (*Coriandrum sativum* L.) from microgreens to mature plant does not seem to undergo radical phenolic transformation, dominated throughout by flavonol glycosides (Barros et al., 2012; El-Zaeddi et al., 2017). The same is true for jute that was shown to maintain a similar phenolic fingerprint high in hydroxycinnamic acids and their derivatives from microgreens (Kyriacou et al., 2019a) to mature plant (Yakoub et al., 2018). It can be therefore hypothesized that the profile and concentration of phenolics in plants during ontogeny depends highly on their genetic constitution, with examples of both interspecific and intraspecific (same species cultivars) already demonstrated (Vallejo et al., 2003; Kyriacou et al., 2019a).

**Table 3:** Phenolic profiles and total phenolic composition of four microgreens genotypes as modulated by variable spectral bandwidths in a controlled growth environment.

Source of variance	Apigenin malonyl glucoside	Caffeic acid	Chlorogenic acid	p-coumaric acid	Ferulic acid	Feruloylquinic acid	Feruloylglycoside	Caffeoyl feruloyl tartaric acid	Kaempferol-3-O-synapoil-sophoroside-7-O-glucoside	Kaempferol-7-O-glucoside	Kaempferolo-3-O-rutinoside	Rutin	Total polyphenols
	(µg g^−1^ dw)	(µg g^−1^ dw)	(µg g^−1^ dw)	(µg g^−1^ dw)	(µg g^−1^ dw)	(µg g^−1^ dw)	(µg g^−1^ dw)	(µg g^−1^ dw)	(µg g^−1^ dw)	(µg g^−1^ dw)	(µg g^−1^ dw)	(µg g^−1^ dw)	(µg g^−1^ dw)
Microgreens species (M)													
Amaranth	4.14 ± 0.1 c	12.78 ± 2.5 bc	9778 ± 12 b	13.8 ± 1.9 c	31.37 ± 3.47 c	970.7 ± 0.27 b	109.3 ± 15.01 b	970 ± 0.04 c	nd	52.55 ± 5.07 a	110.2 ± 11.40 b	1105 ± 124	12825 ± 330 b
Cress	32.86 ± 7.5 b	9.85 ± 1.8 c	998 ± 2 d	47.8 ± 16.8 b	29.29 ± 5.00 b	971.1 ± 0.28 b	129.0 ± 36.66 a	972 ± 0.56 b	9.79 ± 2.1 b	32.29 ± 2.61 d	37.7 ± 8.03 c	1085 ± 152	4354 ± 156 d
Mizuna	61.51 ± 6.3 a	93.77 ± 19.3 a	1112 ± 13 c	89.5 ± 8.9 a	45.36 ± 6.15 a	983.3 ± 1.93 a	72.9 ± 12.35 c	971 ± 0.41 b	1515 ± 154 a	45.42 ± 3.03 b	132.7 ± 30.82 a	nd	5122 ± 212 c
Purslane	36.86 ± 3.7 b	17.11 ± 1.9 b	10125 ± 54 a	8.7 ± 0.7 c	30.10 ± 2.87 b	971.2 ± 0.47 b	61.0 ± 5.21 d	1056 ± 17.62 a	nd	41.42 ± 0.69 c	135.7 ± 7.79 a	1098 ± 107	13581 ± 182 a
Light quality (L)													
Blue	38.73 ± 9.1 a	37.10 ± 9.6 b	5527 ± 1355 a	62.2 ± 15.2 a	43.62 ± 4.30 a	974.7 ± 1.52 a	128.3 ± 24.95 a	1007 ± 18.14 a	874.56 ± 385 a	49.52 ± 5.16 a	149.8 ± 20.59 a	1060 ± 92 b	9250 ± 1320 b
Red	41.11 ± 6.9 a	50.25 ± 19.4 a	5546 ± 1348 a	26.0 ± 5.4 c	35.57 ± 3.57 b	975.3 ± 2.10 a	111.7 ± 10.30 b	995 ± 12.49 b	944.52 ± 422 a	42.00 ± 0.44 b	103.8 ± 12.93 b	1496 ± 61 a	9521 ± 1293 a
Blue-Red	21.68 ± 4.7 b	12.78 ± 2.9 c	5436 ± 1323 b	31.7 ± 12.4 b	20.09 ± 0.47 c	972.1 ± 1.65 b	39.1 ± 4.53 c	977 ± 3.54 c	467.92 ± 207 b	37.25 ± 1.96 c	58.6 ± 10.71 c	732 ± 31 c	8140 ± 1237 c
M × L													
Amaranth × B	4.35 ± 0.1 e	22.41 ± 1.0 cd	9803 ± 5 c	16.1 ± 0.4 de	23.60 ± 0.00 e	971.1 ± 0.27	100.8 ± 4.31 cd	971 ± 0.03 d	nd	72.81 ± 0.31 a	118.9 ± 8.40 d	1389 ± 39 c	13493 ± 37 c
Amaranth × R	4.38 ± 0.2 e	10.04 ± 0.2 efg	9798 ± 6 c	19.1 ± 0.5 d	39.14 ± 0.06 c	971.3 ± 0.14	164.6 ± 4.71 b	970 ± 0.06 d	nd	42.84 ± 0.16 c	143.6 ± 1.13 c	1309 ± 21 c	13473 ± 26 c
Amaranth × B-R	3.68 ± 0.0 e	5.88 ± 0.1 g	9734 ± 6 d	6.3 ± 0.1 e	nd	969.7 ± 0.07	62.5 ± 0.03 e	nd	nd	42.00 ± 0.20 cd	68.0 ± 1.79 f	616 ± 24 f	11508 ± 29 e
Cress × B	23.02 ± 1.5 d	17.02 ± 1.2 def	1002 ± 1 g	114.3 ± 7.1 a	49.28 ± 0.33 b	972.1 ± 0.35	268.6 ± 15.15 a	974 ± 0.28 d	18.03 ± 0.3 d	27.40 ± 0.52 f	66.4 ± 3.41 f	766 ± 13 e	4297 ± 16 h
Cress × R	62.37 ± 1.4 b	6.67 ± 0.4 fg	999 ± 3 g	17.8 ± 1.0 de	19.72 ± 0.04 f	970.7 ± 0.04	94.5 ± 6.50 d	972 ± 0.10 d	6.17 ± 0.1 d	42.69 ± 0.48 c	35.3 ± 0.30 h	1689 ± 55 a	4916 ± 61 g
Cress × B-R	13.18 ± 0.6 e	5.85 ± 0.0 g	992 ± 3 g	11.4 ± 0.8 de	18.87 ± 0.55 f	970.4 ± 0.05	24.0 ± 1.07 g	970 ± 0.11 d	5.16 ± 0.1 d	26.78 ± 0.50 f	11.3 ± 0.42 i	800 ± 25 e	3849 ± 23 i
Mizuna × B	84.32 ± 7.5 a	91.19 ± 8.3 b	1070 ± 8 f	109.9 ± 7.0 ab	62.28 ± 2.54 a	983.3 ± 0.66	77.5 ± 3.60 e	972 ± 0.13 d	1731 ± 88.5 b	56.92 ± 1.59 b	251.6 ± 5.54 a	nd	5491 ± 93 f
Mizuna × R	54.58 ± 1.6 b	160.92 ± 7.9 a	1160 ± 5 e	56.2 ± 3.6 c	52.00 ± 2.06 b	987.3 ± 0.52	112.8 ± 4.25 c	972 ± 0.07 d	1883 ± 116 a	42.37 ± 0.98 cd	100.7 ± 3.42 e	nd	5581 ± 109 f
Mizuna × B-R	45.64 ± 1.5 c	29.22 ± 0.9 c	1106 ± 5 f	102.5 ± 5.7 b	21.81 ± 0.23 ef	979.1 ± 5.19	28.4 ± 1.38 fg	969 ± 0.01 d	931 ± 37.7 c	36.97 ± 0.32 e	45.8 ± 1.58 g	nd	4295 ± 30 h
Purslane × B	43.23 ± 2.2 c	17.79 ± 0.2 de	10233 ± 24 a	8.6 ± 0.1 de	39.31 ± 0.05 c	972.3 ± 0.38	66.3 ± 1.26 e	1111 ± 5.61 a	nd	40.94 ± 1.56 cd	162.4 ± 0.91 b	1024 ± 41 d	13719 ± 56 b
Purslane × R	43.11 ± 6.0 c	23.37 ± 0.9 cd	10227 ± 40 a	11.0 ± 0.1 de	31.42 ± 0.02 d	971.9 ± 0.22	74.9 ± 4.95 e	1067 ± 3.56 b	nd	40.07 ± 0.67 d	135.6 ± 3.95 c	1490 ± 75 b	14115 ± 116 a
Purslane × B-R	24.22 ± 2.0 d	10.18 ± 0.1 efg	9914 ± 3 b	6.5 ± 0.1 e	19.59 ± 0.02 f	969.4 ± 0.03	41.6 ± 0.55 f	991 ± 0.96 c	nd	43.25 ± 0.43 c	109.1 ± 0.61 de	780 ± 14 e	12908 ± 15 d
Significance													
Microgreens species (M)	***	***	***	***	***	***	***	***	***	***	***	ns	***
Light quality (L)	***	***	***	***	***	*	***	***	***	***	***	***	***
M × L	***	***	***	***	***	ns	***	***	***	***	***	***	***

All data are expressed as mean ± standard error, n = 3.

ns, *, *** Nonsignificant or significant at P ≤ 0.05 and 0.001, respectively. Different letters within each column indicate significant differences according to Duncan's multiple-range test (P = 0.05), nd: not detected.

Aside from the genotypic factor, significant differences in the microgreens phenolic composition were also observed in response to light treatments (**Table 3**). A significant M × L interaction characterized variation in almost all the phenolic components quantified, including the total phenolic content. These interactions however stemmed mostly from changes in scale rather than changes in species rank with respect to their response to light treatments. The general response pattern observed across species was a decrease in the concentration of individual phenolic compounds and the total phenolic content when microgreens were grown under combined blue-red bandwidths compared to either bandwidth alone. This decline was particularly sharp in the concentrations of flavonol glycosides, amounting to 67.4%, 48.6%, 53.8%, and 42.7% decrease in feruloylglycoside, kaempferol-3-*O*-synapoil-sophoroside-7-*O*-glucoside, kaempferolo-3-*O*-rutinoside, and rutin, respectively, compared to the mean respective concentrations observed under blue and red light (**Table 3**). The total phenolic content under blue-red light declined by 13.3% compared to the mean content observed under blue and red light.

Previous works have pointed to the influential role of monochromatic blue and red lights, compared to white light, on the synthesis and accumulation of secondary metabolites (Wu et al., 2007; Li and Kubota, 2009; Alrifai et al., 2019). The concerted activation of photoreceptors by LED lights has been hypothesized to influence the up- or down-regulation of enzyme activities responsible for the biosynthesis of PSMs, many of which might constitute key bioactive components (Qian et al., 2016; Alrifai et al., 2019). Different spectral bandwidths were found variably influential on the synthesis, accumulation, and biodegradation of bioactive compounds in lettuce and on chloroplast configuration and photosynthetic performance (Muneer et al., 2014). The current findings are intriguing as they highlight the potential biostimulatory role of blue light, which is sensed by cryptochromes (CRY1 to CRY3) and implicated according to previous studies in the biosynthesis of anthocyanins, chrlorophyll, and carotenoids (Li and Kubota, 2009; Olle and Viršilė, 2013). Although the same researchers supported that red light, sensed by phytochromes (PhyA to PhyE) is chiefly responsible for the synthesis of phenolics, the current work indicates that blue light alone can support the synthesis of phenolic compounds. Targeted increase of flavonol levels by postharvest exposure to blue light has been previously demonstrated on onion bulbs (Ko et al., 2015). Similarly, Taulavuori et al. (2013) reported a 662% and 96% increase in chlorogenic acid content of basil and tomato, respectively, when grown under supplemental blue light vs. HPS light alone. However, significant differences among species as regards their sensitivity to blue light have been reported (Snowden, 2015). The presence of energy-rich shorter wavelengths alone might elicit the increased biosynthesis of flavonoids in order to quench the higher photo-oxidation potential generated. Recent work has demonstrated that blue light may exert oxidative stress in *Arabidopsis* potentially expressed as damage to photosynthetic apparatus, DNA, lipids, and proteins in ways previously seen in response to UV radiation (El-Esawi et al., 2017). Corroborating evidence from recent works suggests that blue light elicits the biosynthesis of phenolic acids and flavonoids in plants by triggering the expression of key enzymes in the phenylpropanoid pathway in order to counteract oxidative stress, although the species-specificity underscoring these transcriptional responses remains under-characterized and poorly understood (Kojima et al., 2010; Huché-Thélier et al., 2016; Hasan et al., 2017; Kitazaki et al., 2018; Taulavuori et al., 2018). Experiments with *Arabidopsis* photoreceptor mutants have confirmed that blue light-mediated CRY photoreception systems control chalcone synthase expression (Jenkins et al., 2001). Blue light's species-specific action on CRY gene expression seems key to unraveling plant species-specific responses to monochromatic light, (Huché-Thélier et al., 2016).

It is nonetheless intriguing why the simultaneous exposure of microgreens to short (blue) and long (red) wavelengths compromises the effects of either bandwidth alone. It is possible that exposure to both bandwidths might promote the photoisomerization of phenolic compounds thus complicating the quantification process (Saltveit, 2010). The efficacy of light-induced effects in conferring physiological and compositional changes to plants, including the stimulation of the phenylpropanoid pathway, has been previously demonstrated; however, the nature of these effects is contested between supporters of epigenetic mechanisms and those proposing changes in the PS II photochemistry mediated by redox signaling in the photosynthetic apparatus (Ganguly et al., 2018; Sytar et al., 2019).

### Principle Component Analysis of Polyphenolic Composition of Microgreen Species Grown Under Variable Bandwidths

**Figure 3:**
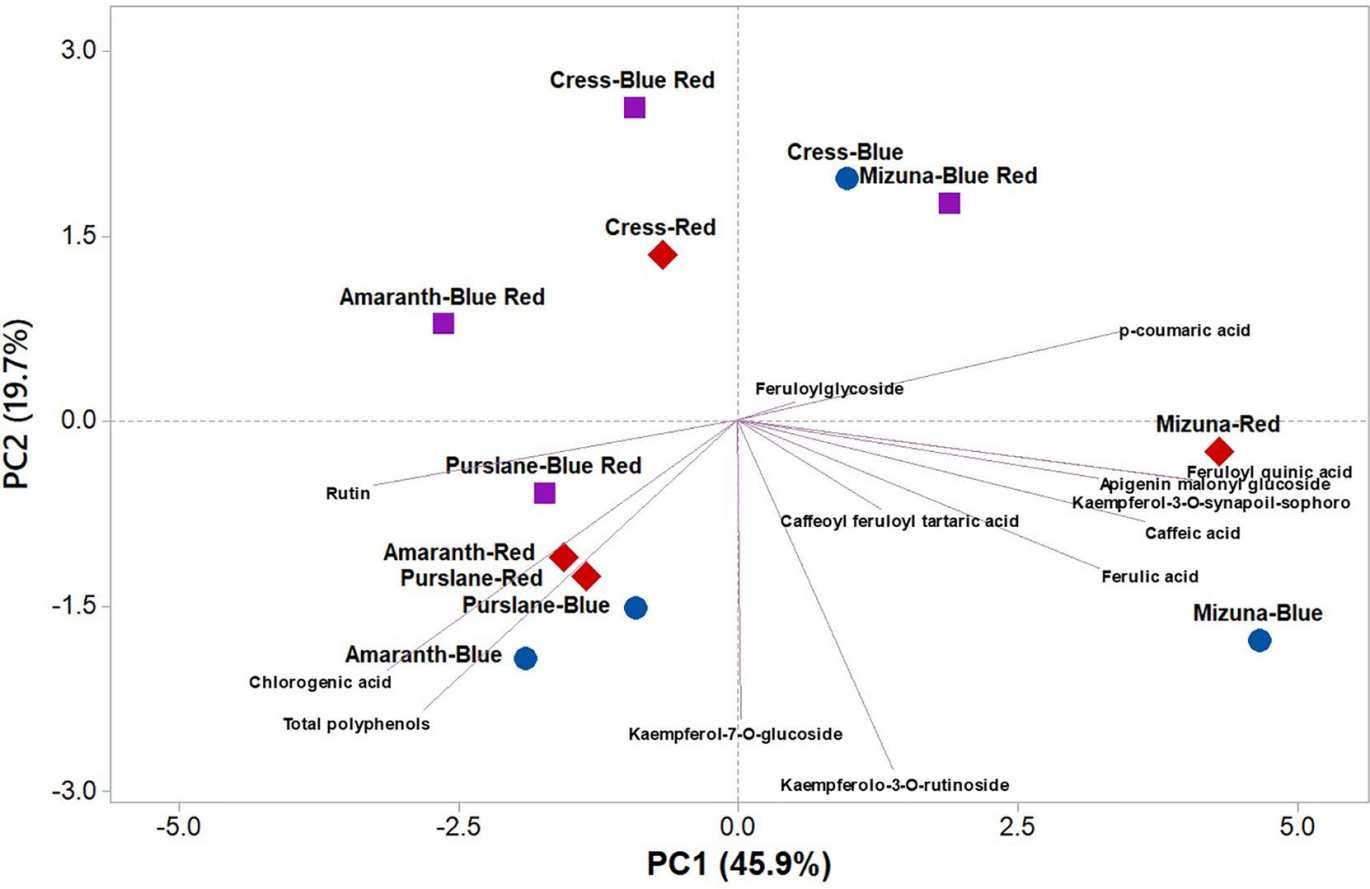
Principal component loading plot and scores of principal component analysis (PCA) of the concentrations of 13 key phenolic compounds and total polyphenols identified and quantitated by UHPLC-Q-Orbitrap HRMS analysis in four microgreens genotypes as modulated by variable spectral bandwidths in a controlled growth environment.

PCA was performed on polyphenolic profile and on total phenolic content of microgreens species grown under variable bandwidths (**Figure 3**). The first three principal components (PCs) were related with eigen values higher than one and explained 79.2% of the total variance, with PC1, PC2, and PC3 accounting for 45.2%, 22.0%, and 12.0%, respectively (**Supplementary Table S1**). The first PC was positively correlated to kaempferol-3-*O*-synapoil-sophoroside-7-*O*-glucoside, feruloyl quinic acid, caffeic acid, p-coumaric acid, ferulic acid, and apigenin malonyl glucoside (**Supplementary Table S1**). PC1 was also negatively correlated to rutin, chlorogenic acid and total polyphenols. Moreover, PC2 was positively correlated to kaempferolo-3-*O*-rutinoside, kaempferol-7-*O*-glucoside, and total polyphenols (**Supplementary Table S1**). Furthermore, the loading matrix indicates the correlations among the examined 13 key phenolic compounds and total phenolic content. In the current study for instance, variation in chlorogenic acid and caffeoyl feruloyl tartaric acid was most closely aligned with total polyphenols content, whereas variation in p-coumaric acid was not correlated to total phenolics (**Figure 3**).

The score plot of the PCA integrated useful information on the polyphenolic profile of the tested microgreens genotypes grown under monochromatic red or blue, or dichromatic red and blue LED lighting. The mizuna microgreen grown under blue and to a lesser extent under red LED lighting was positioned on the positive side of PC1 in the lower right quadrant of the PCA score plot as it delivered microgreens of premium quality with high concentration of apigenin malonyl glucoside, caffeic acid, ferulic acid, and kaempferolo-3-*O*-rutinoside (**Figure 3**). Moreover, purslane grown under monochromatic blue or red LED lighting was positioned in the lower left quadrant, characterized overall by higher chlorogenic acid, rutin, and total polyphenols (**Figure 3**).

### Principle Component Analysis of Growth Parameters, Mineral Profile, and Nutritional and Functional Traits of Microgreen Species Grown Under Variable Bandwidths

**Figure 4:**
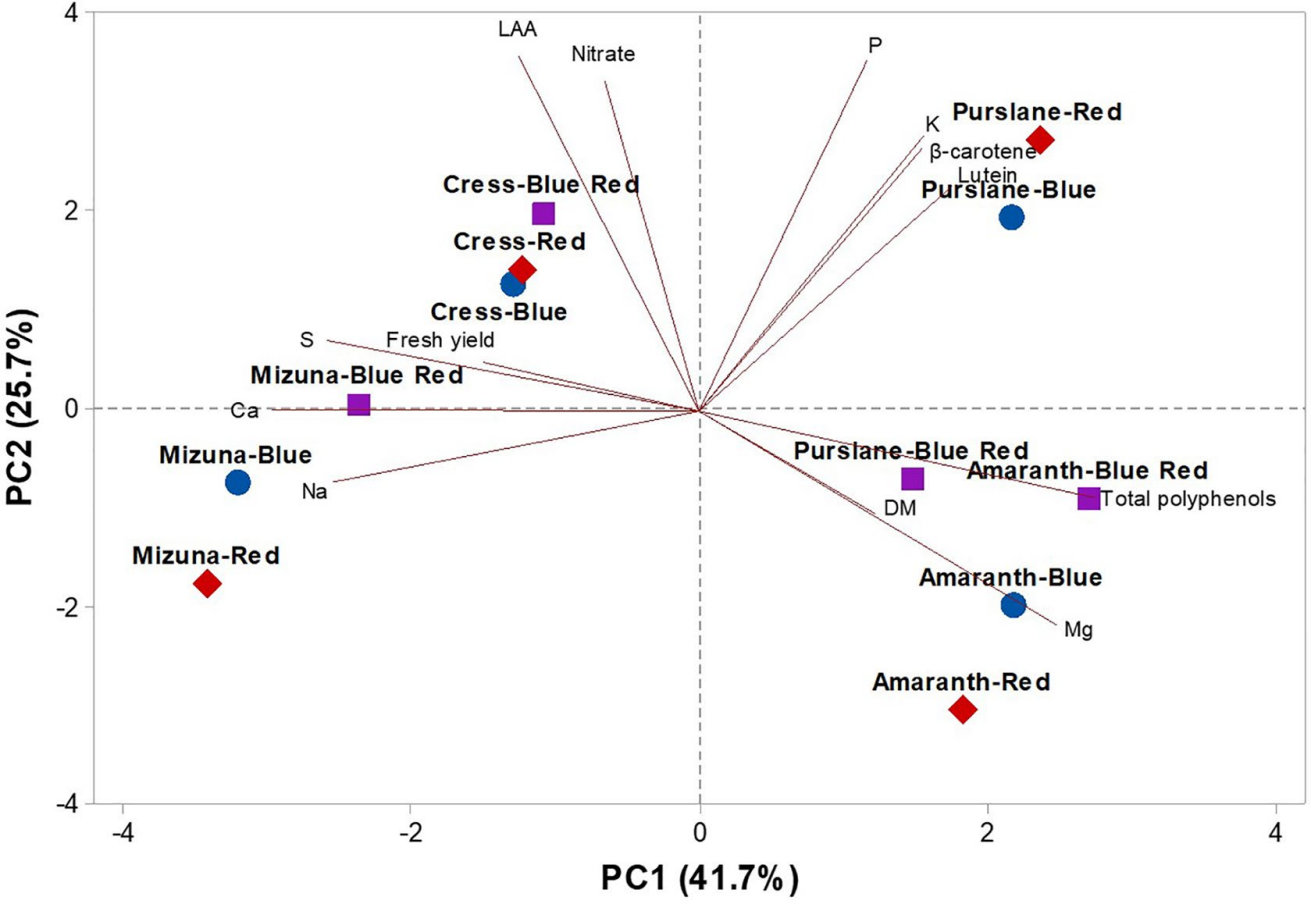
Principal component loading plot and scores of PCA fresh yield and dry matter content, mineral concentrations (nitrate, P, K, S, Ca, Mg, and Na), lipophilic antioxidant activity (LAA), lutein, β-carotene, and total phenolics concentrations in four microgreens genotypes as modulated by variable spectral bandwidths in a controlled growth environment.

To obtain an in-depth overview of productivity, mineral profile, nutritional and functional traits of the four microgreens genotypes as modulated by variable spectral bandwidths, a second PCA was performed on fresh yield, dry matter, mineral composition (nitrate, P, K, Ca, Mg, S, and Na), carotenoid composition (lutein and β-carotene), and total polyphenols (**Figure 4**). The first three PCs were related with eigen values > 1 and explained 85.5% of the total variance, with PC1, PC2, and PC3 accounting for 41.7%, 25.7%, and 18.1%, respectively (**Supplementary Table S2**). Furthermore, the loading matrix indicates the correlations among the examined quanti-qualitative traits. In our study, we discerned four groups of positively correlated variables (**Figure 4**): i) the group in the upper left quadrant comprising nitrate and lipophilic antioxidant activity, ii) the group in the upper right quadrant comprising the two carotenoids, P, and K, iii) the group clustered in the lower right quadrant comprising total polyphenols and Mg and finally, and iv) the group in the lower left quadrant comprising fresh yield and most of the mineral composition (S, Ca, and Na; **Figure 4**).

The effectiveness of PCA in interpreting genotype differences across multiple nutritional and functional quality characters in response to several pre-harvest factors (e.g., light conditions, nutrient solution management, and biofortification) has been previously demonstrated (Colonna et al., 2016; Cardarelli et al., 2017; Rouphael et al., 2017a; Rouphael et al., 2017b; El-Nakhel et al., 2019; Kyriacou et al., 2019a; Rouphael et al., 2019). This was also the case in the current experiment, since the score plot of the PCA highlighted crucial information on the nutritional and functional quality of the tested microgreens genotypes exposed to variable bandwidths. The PCA clearly divided the four tested microgreens along PC1 with *Brassicaceae* microgreens (cress and mizuna) on the negative side and amaranth and purslane microgreens on the positive side (**Figure 4**). Accordingly, *Brassicaceae* microgreens distinguished for higher fresh yield, nitrate, and mineral profile (S and Ca contents); whereas the amaranth and purslane were superior in target lipophilic antioxidant molecules as well as in total polyphenols (**Figure 4**). Particularly, the purslane grown under monochromatic red LED lighting, positioned in the upper right quadrant of the PCA score plot, delivered premium quality and high concentration of lipophilic antioxidants such as lutein and β-carotene and P content (**Figure 4**). Moreover, cress grown under monochromatic and dichromatic red and blue LED lighting was positioned in the upper left quadrant, characterized overall by higher lipophilic antioxidant activity and nitrate content. Finally, the lower left quadrant depicted treatments (mizuna grown under red/blue/blue-red LED lighting) characterized by high fresh yield, and Ca and S contents (**Figure 4**). The PCA performed in the present experiment configured an integrated view of fresh yield and quality traits quantified by ion chromatography, HPLC-DAD and UHPLC-Q-Orbitrap HRMS. Thus it enabled the interpretation of variation patterns in these traits with respect to the microgreens genotypes and the studied select spectral bandwidths.

## Conclusions

Targeted modulation of microgreens secondary metabolism through select spectral bandwidths is presently assessed as a tool to produce phytochemically-enriched microgreens of high functional quality and nutritive value. Growth and yield parameters dependent on primary metabolism were optimized under dichromatic blue-red light. Nitrogen assimilation mediated by nitrate and nitrite reduction was hampered under monochromatic blue and red lights but was more efficiently promoted by dichromatic blue-red light. Therefore, the use of monochromatic blue or red light as opposed to combined blue-red bandwidths may result in higher accumulation of nitrate in microgreens. Spectral effects seemed less consistent with respect to microgreens' mineral composition, variation in which was primarily genotypic. Concentrations of key carotenoids lutein and β-carotene and the lipophilic antioxidant capacity of microgreens were favored by blue-red light. Purslane however diverged from this response pattern, highlighting the underlying genotype specificity of these responses that requires additional investigation. Analysis of polyphenols by Orbitrap LC-MS/MS revealed substantial genotypic differences with respect to composition. The general response across species was a decrease in individual polyphenolic constituents, particularly flavonol glycosides, and total polyphenols under blue-red light. The current work highlights how select genetic background combined with effective light management might facilitate production of microgreens with superior functional quality.

## Data Availability Statement

The datasets generated for this study are available on request to the corresponding author.

## Author Contributions

MK and YR coordinated the whole project, provided the intellectual input and set up the experiment. YR wrote the introduction and the PCA sections. MK wrote the rest of the manuscript. CE-N, AP, GS, and MG were involved in data analysis and data interpretation. GG performed the HPLC-DAD and Orbitrap LC-MS/MS analysis. AZ, AR, and SP revised the manuscript.

## Funding

The work was supported by the Italian Space Agency through the project "Microgreens × Microgravity (MICRO×2)."

## Conflict of Interest

The authors declare that the research was conducted in the absence of any commercial or financial relationships that could be construed as a potential conflict of interest.
