# Supplementary material for: Genotype-Specific Modulatory Effects of Select Spectral Bandwidths on the Nutritive and Phytochemical Composition of Microgreens
Source: Front Plant Sci. 2019 Nov 19;10:1501. doi: 10.3389/fpls.2019.01501 (PMC6896982; doi:10.3389/fpls.2019.01501)
Supplement: Supplementary file 1 [file Table_1.doc]

**Supplementray Table 1.** Eigen values, relative and cumulative proportion of total variance, and correlation coefficients for polyphenols profile in four microgreens genotypes as modulated by variable spectral bandwidths with respect to the three principal components.

| Principal components | PC1 | PC2 | PC3 |
| --- | --- | --- | --- |
| Eigen value | 5.8 | 2.8 | 1.5 |
| Percentage of variance | 45.2 | 22.0 | 11.9 |
| Cumulative variance | 45.2 | 67.2 | 79.1 |
|  |  |  |  |
| Eigen vectors ^a^ |  |  |  |
| Kaempferol-3-O-synapoil-sophoroside-7-O-glucoside sophoroside-7-O-glucosidesophoroside-7-O-glucoside | **0.943** | 0.218 | -0.190 |
| Feruloyl quinic acid | **0.932** | 0.222 | -0.140 |
| Rutin | **-0.849** | 0.035 | 0.171 |
| Caffeic acid | **0.826** | 0.317 | -0.086 |
| p-coumaric acid | **0.788** | -0.200 | 0.345 |
| Chlorogenic acid | **-0.724** | 0.606 | 0.044 |
| Ferulic acid | **0.684** | 0.436 | 0.517 |
| Apigenin malonyl glucoside | **0.672** | 0.222 | -0.303 |
| Kaempferolo-3-O-rutinoside | 0.233 | **0.893** | 0.174 |
| Total polyphenols | **-0.669** | **0.704** | 0.054 |
| Feruloylglycoside | 0.086 | -0.104 | **0.964** |
| Caffeoyl feruloyl tartaric acid | -0.375 | 0.525 | -0.123 |
| Kaempferol-7-O-glucoside | -0.009 | **0.635** | -0.081 |
|  |  |  |  |

^a^Boldface factor loadings are considered highly weighed

**Supplementary Table 2.** Eigen values, relative and cumulative proportion of total variance, and correlation coefficients for growth parameters, mineral profile, nutritional and functional traits in four microgreens genotypes as modulated by variable spectral bandwidths with respect to the three principal components.

| Principal components | PC1 | PC2 | PC3 |
| --- | --- | --- | --- |
| Eigen value | 5.4 | 3.3 | 2.3 |
| Percentage of variance | 41.7 | 25.7 | 18.1 |
| Cumulative variance | 41.7 | 67.4 | 85.5 |
|  |  |  |  |
| Eigen vectors ^a^ |  |  |  |
| Ca | **-0.971** | 0.004 | -0.034 |
| Total polyphenols | **0.900** | -0.199 | 0.317 |
| S | **-0.846** | 0.164 | -0.483 |
| Na | **-0.830** | -0.162 | 0.472 |
| Mg | **0.812** | -0.492 | 0.129 |
| LAA | -0.410 | **0.818** | 0.041 |
| P | 0.381 | **0.806** | 0.163 |
| Nitrate | -0.215 | **0.761** | -0.499 |
| K | 0.510 | **0.634** | 0.220 |
| β-carotene | 0.506 | **0.604** | 0.106 |
| DM | 0.401 | -0.237 | **-0.854** |
| Fresh yield | -0.489 | 0.111 | **0.845** |
| Lutein | **0.600** | 0.518 | -0.004 |
|  |  |  |  |

^a^Boldface factor loadings are considered highly weighed

^b^ LAA, Lipophylic antioxidant activity; DM, dry matter.
